# Dual pathology in aortic stenosis: Identifying transthyretin amyloidosis likelihood in patients referred for transcatheter aortic valve implantation

**DOI:** 10.1016/j.ijcha.2026.101991

**Published:** 2026-08-12

**Authors:** Stéphanie Kristina Schwarting, Selen Alieva, Mohammad Usama Chaudhry, Kornelia Löw, Sergio Grosu, Steffen Massberg, Simon Deseive, Julius Steffen

**Affiliations:** a Department of Medicine I, LMU University Hospital Munich, Ludwig-Maximilians-University Munich, Marchioninistrasse 15, Munich, Germany; b German Center for Cardiovascular Research DZHK e.V., Munich Heart Alliance, Munich, Germany; c Department of Cardiology, Angiology and Respiratory Medicine, University Hospital Heidelberg, Im Neuenheimer Feld 410, Heidelberg, Germany; d Department of Radiology, LMU University Hospital Munich, Marchioninistrasse 15, Munich, Germany

**Keywords:** Cardiac amyloidosis, Aortic stenosis, Flow groups, Dual pathology

## Abstract

**Background:**

Aortic stenosis (AS) and transthyretin cardiac amyloidosis (CA) may coexist with overlapping clinical and imaging features. Data on the prevalence of this dual pathology (AS-CA) across AS flow groups are limited. This study assessed the likelihood of AS-CA in different AS flow groups and its association with outcomes after transcatheter aortic valve implantation (TAVI).

**Methods:**

We retrospectively analyzed 2764 patients (median age 82 years, 55% male) undergoing TAVI for severe native AS between 2017 and 2022). High likelihood of AS-CA was defined as RAISE score ≥ 3 points together with a T-AMYLO score ≥ 3 + red-flag criteria, or a T-AMYLO score ≥ 7 points. Procedural outcomes and long-term survival were evaluated.

**Results:**

High AS-CA likelihood was identified in 235 patients (9%). The estimated likelihood was comparable across AS flow groups, including high-gradient (7.4%), classical low-flow low-gradient (10.6%), paradoxical low-flow low-gradient (9.9%), and normal-flow high-gradient AS patients (7.7%) (*p* = 0.09). Procedural success after TAVI did not differ between patients with AS-CA and lone AS. However, AS-CA likelihood was associated with a higher 3-year all-cause mortality (44% vs. 29%, *p* < 0.001). This association was attenuated after adjustment for Society of Thoracic Surgeons (STS) risk score (HR 1.2 [95% CI 0.99–1.53], *p* = 0.065).

**Conclusion:**

Dual pathology of AS-CA is likely present in approximately 9% of TAVI patients and is not confined to specific AS flow groups. Although procedural outcomes are comparable, AS-CA is associated with increased mortality, largely driven by baseline risk. Further studies are needed to improve diagnostic strategies and clarify the underlying mechanisms.

## 1 Introduction

Patients with aortic stenosis (AS) may also have concomitant transthyretin amyloid cardiomyopathy (CA) since symptoms and echocardiographic findings overlap. Different likelihood scores such as RAISE (**R**emodeling, **A**ge, cardiac **I**njury, **S**ystemic and **E**lectrical abnormalities) and the simplified T-AMYLO (carpal **T**unnel syndrome, **A**ge, **M**ale gender, h**Y**pertrophy, **LO**w QRS voltage) have been proposed to help identify CA in AS patients. [1], [2] Prevalence of the dual pathology AS-CA is thought to range from 8 to 16% of AS patients, and transcatheter aortic valve implantation (TAVI) may have positive implications on the course and outcome of the coexistent cardiac disease. [3], [4]

Patients with AS are commonly subdivided into four different flow groups: high-gradient (HG; mean pressure gradient (dpmean) ≥40 mmHg), classical low-flow, low-gradient (cLFLG, dpmean <40 mmHg, left ventricular ejection fraction (LVEF) <50%), paradoxical low-flow, low-gradient (pLFLG, dpmean <40 mmHg, LVEF ≥50%, stroke volume index (SVi) ≤35 ml/m^2^), and normal flow, low-gradient (NFLG, dpmean <40 mmHg, LVEF ≥50%, SVi >35 ml/m^2^). While there is strong evidence supporting the therapeutic benefits from TAVI in HG or cLFLG patients, the evidence is less robust for those with pLFLG and NFLG. It is commonly hypothesized that CA is a comorbidity particularly among pLFLG patients. [5]

The purpose of this single-center study was to assess likelihood of dual pathology (AS-CA) across different aortic stenosis flow groups according to screening score results and to evaluate periprocedural success and long-term mortality after TAVI in patients with likely AS-CA.

## 2 Methods

**Fig. 1:**
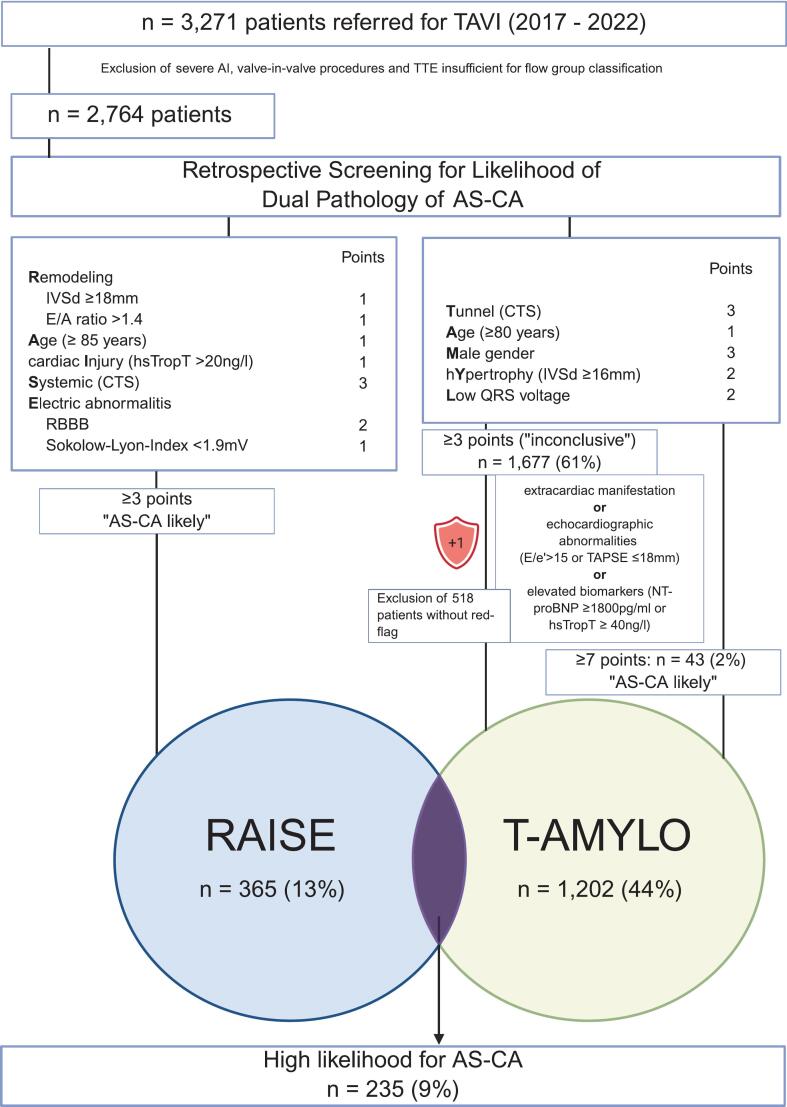
Likelihood of dual pathology of aortic stenosis and cardiac amyloidosis was analyzed in a cohort of 2764 patients referred for TAVI by applying the two scores RAISE (Remodeling, Age, cardiac Injury, Systemic and Electrical abnormalities) and T-AMYLO (carpal Tunnel syndrome, Age, Male gender, hYpertrophy, LOw QRS voltage). High likelihood of dual pathology AS-CA was defined as meeting thresholds in both scores. AS: aortic stenosis; CA: cardiac amyloidosis; CTS: carpal tunnel syndrome; hsTropT: high-sensitivity troponin T; NT-proBNP: N-terminal pro brain-natriuretic peptide; RBBB: complete right bundle branch block; TAPSE: tricuspid annular plane systolic excursion; TAVI: transcatheter aortic valve implantation.

Between 2017 and 2022, 3271 patients were referred for TAVI at our center based on routine clinical practice and guideline recommendations, without additional referral pathways or predefined selection criteria beyond standard eligibility criteria. Of these, 507 patients were excluded due to severe aortic regurgitation, valve-in-valve procedures, or echocardiographic data deemed insufficient for flow group classification (Fig. 1). A total of 2764 consecutive patients with native severe AS were included in the retrospective evaluation of dual likelihood for AS-CA.

Score-specific cut-offs for likely CA were defined as ≥3 points for the RAISE score [1] and ≥ 7 points or ≥ 3 plus at least one additional red-flag criterion for the T-AMYLO score [2]. In the present analysis, red-flag criteria included extracardiac manifestations (excluding carpal tunnel syndrome, already present in the main score), echocardiographic abnormalities (E/e’ > 15 or TAPSE ≤18 mm), or elevated biomarkers, defined as N-terminal pro B-type natriuretic peptide (NT-proBNP) ≥1800 pg/ml or high-sensitive troponin T (hsTnT) ≥40 ng/ml (Fig. 1). The selection of these red-flag criteria was based on variables that demonstrated significant differences between patients with and without ATTR-CM in the original derivation cohort. [2] Diagnostic performances of both prediction models were previously described with an AUC of 0.85 [95%CI: 0.79–0.91] for the RAISE score and an AUC (in AS group) of 0.85 [95%CI: 0.77–0.93] for the T-AMYLO score, respectively. [1], [2] Sensitivity and specificity of both scores vary significantly. While the RAISE score favors sensitivity (94%) (specificity of 52%), the T-AMYLO score favors specificity (97%) (sensitivity of 37%). Given the different diagnostic characteristics of the RAISE and T-AMYLO score, concordant positivity of both scores was required to provide a conservative estimate of AS-CA likelihood while reducing dependence on a single screening tool. This approach was expected to increase specificity at the expense of sensitivity in order to make our analysis more robust.

Combined Valve Academic Research Consortium 3 (VARC-3) endpoints and 3-year all-cause mortality following TAVI were analyzed, and mortality analysis was adjusted for the Society of Thoracic Surgeons (STS) risk score. All data were collected as part of the EVERY-VALVE registry as described before. [6] The study complied with the 1975 Declaration of Helsinki; data analysis was approved by the local ethics committee (number 19–840).

Numeric data were tested for normal distribution using Shapiro-Wilk test and are presented as median [inter-quartile range] or mean ± standard deviation. Data were compared using Kruskal-Wallis test, Fisher's exact test, or Chi-squared test as appropriate. Mortality rates were compared using log-rank test and graphically presented using Kaplan Meier estimates. Bonferroni-method was applied to account for multiple testing. A *p*-value <0.05 was considered significant. Calculations were performed with R (R Foundation for Statistical Computing, Vienna, Austria, version 4.3.2). BioRender (BioRender.com), Prism (GraphPad Prism for macOS, version 11.0.0, Boston, MA, USA) and Affinity Designer 2 (Serif, Europe, version 2.6.2) were used for illustrative content.

## 3 Results

In the overall cohort, median age was 82 years (interquartile range [IQR], 78–85 years), with 55% men. The distribution across AS flow groups was as follows: 1309 (47%) HG, 498 (18%) cLFLG, 517 (19%) pLFLG, and 440 (16%) NFLG AS patients. Clinical characteristics were consistent with the typical subgroups (Supplementary Table 1). [6]

Likelihood scores estimated an overall presence of AS-CA in 13% (RAISE), and 44% (T-AMYLO), respectively. Achievement of the RAISE score-specific cut-off was primarily driven by three criteria: age ≥ 85 (1 point), the presence of right bundle branch block (2 points) and elevated hsTnT (1 point), the latter being fulfilled by more than 50% of all patients. In the T-AMYLO score, main drivers were male sex (3 points) and age ≥ 80 (1 point) (Supplementary Fig. 1).

**Fig. 2:**
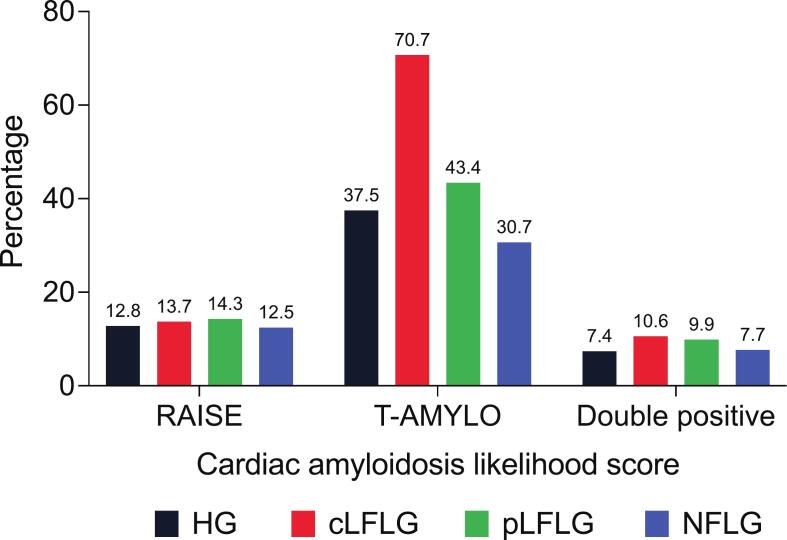
Likelihood of dual pathology according to aortic stenosis (AS) flow group. Distribution of aortic stenosis flow groups (high-gradient [HG] (black bars), classical low-flow low-gradient [cLFLG] (red bars), paradoxical low-flow low gradient [pLFLG] (green bars) and normal flow low-gradient [NFLG] (blue bars)) according to cardiac amyloidosis (CA) likelihood scores. Significant differences in the distribution of AS flow groups were observed for the T-AMYLO score (*p* < 0.001), whereas no significant differences were found for the RAISE score and the combined double-positive classification (*p* = 0.80 and *p* = 0.09, respectively).

235 patients (9%) fulfilled likelihood criteria in both scores. They were significantly older, had a higher Society of Thoracic Surgeon (STS) score and more cardiovascular comorbidities than AS lone patients (Table 1). Cardiac biomarkers, including NT-proBNP and hsTnT, were significantly higher in patients with likely AS-CA. Echocardiography demonstrated significant differences in left ventricular ejection fraction (LVEF), wall thickness parameters and aortic valve pressure gradients (maximum and mean) compared with patients with lone AS. Stroke volume index (SVi) and right ventricular functional parameters were also significantly lower in the likely AS-CA group (Table 1). Likelihood of dual pathology was similarly high within each flow group (HG 7.4%, cLFLG 10.6%, pLFLG 9.9%, and NFLG 7.7%, respectively, *p* = 0.09), although both scores showed distinct distributions across flow groups when considered individually, particularly with a higher rate of T-AMYLO score positivity in patients with cLFLG (Fig. 2).

**Table 1:** Characteristics of patients with high likelihood of dual pathology of aortic stenosis and cardiac amyloidosis (AS-CA).

	AS lone (*n* = 2529)	AS-CA likely (*n* = 235)	*p* value
RAISE score	1 [0–2]	3 [3–4]	<0.01
T-AMYLO score	3 [1–4]	4 [4–6]	<0.01
Male sex, n	1359 (54)	172 (73)	<0.01
Age, years	81.5 [77.2–85.0]	85.5 [81.1–87.9]	<0.01
Body mass index, kg/m^2^	25.8 [23.3–29.0]	26.5 [24.1–30.1]	<0.01
STS score, points	3.0 [2.0–5.0]	4.0 [2.7–6.9]	<0.01
Hypertension, n	2260 (90)	221 (94)	0.03
Type 2 DM, n	683 (27)	73 (31)	0.20
Smoker (active or past), n	599 (24)	60 (26)	0.53
COPD, n	197 (13)	11 (8)	0.14
Hypercholesterolemia, n	1303 (52)	111 (48)	0.20

Comorbidities			
CKD, n	936 (37)	126 (54)	<0.01
Creatinine, mg/dl	1.1 [0.9–1.4]	1.3 [1.0–1.6]	<0.01
eGFR, ml/min/1.73m^2^	62.0 (44.8–80.0)	51.0 (33.2–69.8)	<0.01
CAD, n	1432 (57)	154 (66)	<0.01
Prior MI, n	251 (10)	39 (17)	<0.01
Afib/Aflutter, n	422 (17)	68 (29)	<0.01
NTproBNP, pg/ml	1605 [601–4199]	2792 [1198–5909]	0.03
hsTroponin T, ng/l	25.0 (16.0–44.2)	43.5 (28.2, 63.8)	0.81
AS flow groups			0.09
HG	1212 (48)	91 (41)	
cLFLG	445 (18)	53 (23)	
pLFLG	470 (18)	51 (22)	
NFLG	406 (16)	34 (15)	

Echocardiographic parameters			
LVEF, %	55 [50–55]	55 [45–55]	<0.01
LVEF ≥50%, n LVEF 41–49%, n LVEF ≤40%, n	1894 (75) 271 (11) 360 (14)	159 (68) 36 (15) 39 (17)	
IVSd, mm	13 [11–15]	14 [12–17]	<0.01
LVPWd, mm	11 [10−13]	12 [10–14]	<0.01
RWT, %	0.5 [0.4–0.6]	0.5 [0.4–0.6]	<0.01
LVEDd, mm	46 [42–52]	47 [42–53]	0.27
E to A	0.8 [0.6–1.2]	1.3 [0.7–2.1]	<0.01
E/e’	13.9 [10.5–18.2]	16.3 [12.4–21.3]	<0.01
e’	6.2 [5.1–7.7]	6.1 [4.8–7.3]	0.22
AVA, cm^2^	0.7 [0.6–0.8]	0.7 [0.6–0.9]	0.72
AVAi, cm^2^/m^2^	0.4 [0.3–0.5]	0.4 [0.3–0.4]	0.35
Dpmax, mmHg	62 [47–74]	59 [43–70]	<0.01
Dpmean, mmHg	39 [28–47]	36 [26–44]	<0.01
SVi, ml/m^2^	35 [28–42]	32 [26–40]	<0.01
AR grade > 1, n	283 (11)	30 (13)	0.47
RA area, mm^2^	20 [16–25]	22 [18–28]	<0.01
LA area, mm^2^	24 [20–28]	26 [22−31]	<0.01
MR grade ≥ 3, n	99 (4)	9 (4)	0.94
TR grade > 1, n	348 (14)	47 (20)	0.01
TR gradient, mmHg	33 (26–43)	37 (30–43)	0.03
TAPSE, mm	21 [18–24]	19 [16–23]	<0.01
RV-PAc, mm/mmHg	0.8 (0.5–2.8)	0.6 (0.4–2.5)	0.05

Data are presented as *n* (%) or median [IQR]. A *p* value of < 0.05 was considered significant.

Afib/flutter: atrial fibrillation/atrial flutter; AR: aortic regurgitation; AVA: aortic valve area; AVAi: aortic valve area index; dpmax: maximum pressure gradient; dpmean: mean pressure gradient; CAD: coronary artery disease; CKD: chronic kidney disease; COPD: chronic obstructive pulmonary disease; DM: diabetes mellitus; eGFR: estimated glomerular filtration rate; HG: high-gradient; hs Troponin T: high-sensitive troponin T; IVSd: interventricular septum diastolic diameter; LA: left atrium; LFLG: low-flow, low-gradient; LVEF: left-ventricular ejection fraction; LVIDd left ventricular internal diastolic diameter; LVPWd left ventricular posterior wall diastolic diameter; MI: myocardial infarction; MR: mitral regurgitation; NFLG: normal-flow, low-gradient; NT-proBNP: N-terminal pro brain-natriuretic peptide; RA: right atrium; RV-PA: right-ventricular to pulmonary artery coupling; RWT: relative wall thickness; STS-Score: Society of Thoracic Surgeon (STS) score; SV: stroke volume; SVi: stroke volume index; TAPSE; tricuspid annular plane systolic excursion; TR: tricuspid regurgitation.

Although not the primary aim of this study, retrospective evaluation revealed that further diagnostic work-up for cardiac amyloidosis had been undertaken in very few patients. In line with the subsequently established guideline recommendations, this included serum/ urine immunofixation in 17 (7%) and bone scintigraphy in 9 patients (4%) with likely AS-CA according to two positive risk scores, respectively. Diagnostic myocardial tracer uptake (Perugini grade 2–3) was observed in half of the patients undergoing scintigraphy, one patient showed grade 1 uptake. Monoclonal gammopathy was detected in three patients who did not undergo subsequent bone scintigraphy and showed no evidence of light-chain amyloidosis. Abnormal free-light chains or extensive ratio were only observed in these three patients. Overall, ATTR-CM was finally confirmed in only four patients with double positivity in both likelihood scores; in one of these cases, the diagnosis was established by histological confirmation on endomyocardial biopsy.

The VARC-3 combined endpoints technical success (lone AS, 96% and AS-CA, 97%, *p* = 0.40) and device success (lone AS, 89% and AS-CA, 91%, *p* = 0.45) occurred at similar rates without significant differences for any of the components. Importantly, there were no differences in stroke rate or need for pacemaker implantation within 30 days after TAVI (Table 2).

**Table 2:** Combined valve academic research consortium 3 (VARC-3) endpoints technical success and device success and outcomes.

	AS lone (*n* = 2546)	AS-CA (*n* = 216)	p value
VARC-3 endpoint technical failure, n (%)	90 (3.5%)	6 (2.8%)	0.56
Procedural death, n (%)	17 (0.7%)	2 (0.7%)	0.66
Structural complications, n (%)	45 (1.8%)	4 (1.9%)	0.93
Conversion to open surgery, n (%)	11 (0.4%)	0 (0.0%)	0.33
Prosthesis dislocation, n (%)	11 (0.4%)	0 (0.0%)	0.33
Second valve prosthesis required, n (%)	14 (0.5%)	1 (0.5%)	0.30
Device failure VARC-3, n (%)	274 (10.8%)	21 (9.7%)	0.63
Early safety at 30 days VARC-3, n (%)	722 (28.4%)	76 (35.2%)	0.03
All-cause mortality at 30 days, n (%)	60 (2.4%)	9 (4.2%)	0.10
Postprocedural (30 days) elevated dpmean, n (%)	79 (3.1%)	1 (0.5%)	0.03
Vascular interventions at 30 days, n (%)	34 (1.3%)	1 (0.5%)	0.27
Early myocardial infarction at 48 h, n (%)	15 (0.6%)	0 (0.0%)	0.26
Stroke rate at 30 days, n (%)	44 (1.7%)	6 (2.8%)	0.27
Drop of hemoglobin >3 g/dl or transfusion, n (%)	299 (11.7%)	26 (12.0%)	0.90
Acute kidney injury stage 3 or 4, n (%)	12 (0.5%)	2 (1.0%)	0.34
Postprocedural pacemaker implantation at 30 days, n (%)	322 (12.6%)	34 (15.7%)	0.19

Data are presented as *n* (%)]. A *p* value of < 0.05 was considered significant.

VARC-3: Combined Valve Academic Research Consortium 3 endpoint.

**Fig. 3:**
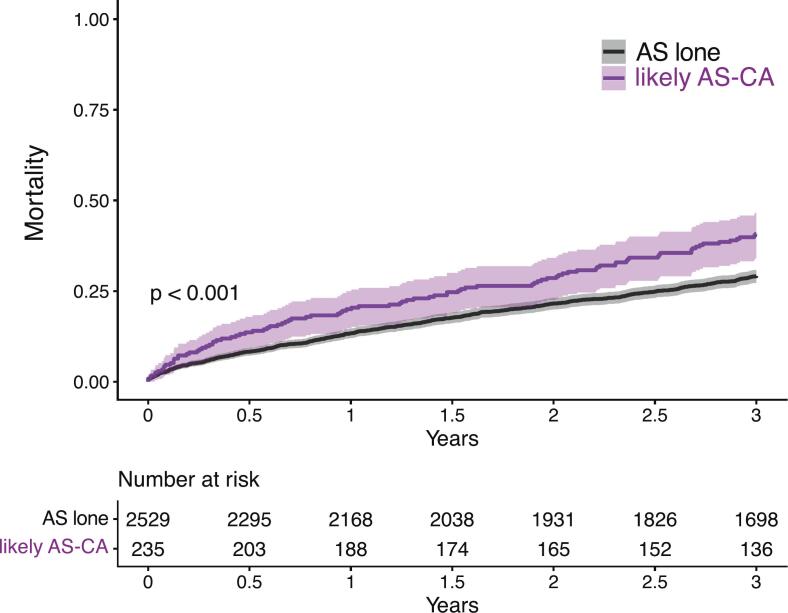
Kaplan-Meier estimates of 3-year all-cause mortality according to AS-CA status. Patients with likely AS-CA exhibited significantly higher mortality compared with those with AS alone over a 3-year follow-up period (log-rank *p* < 0.0001). Adjusted HR was 1.2 (95%CI 0.99–1.53), *p* = 0.065). AS-CA: aortic stenosis and cardiac amyloidosis.

**Fig. 4:**
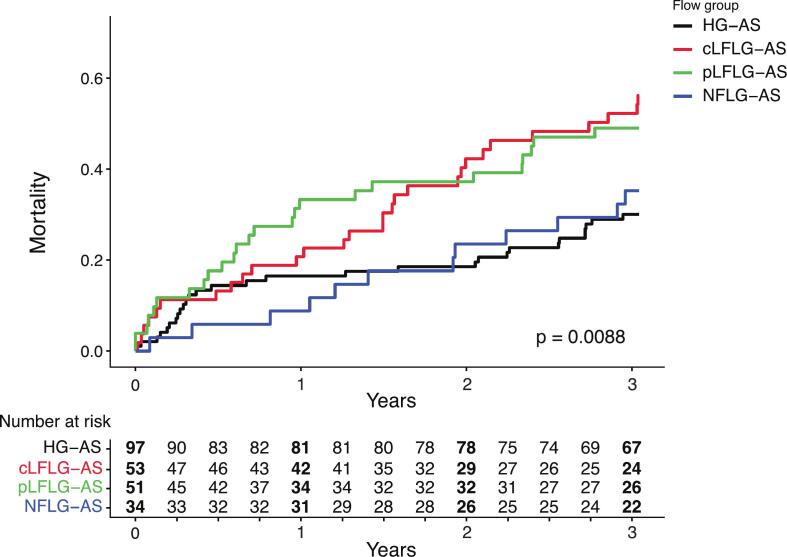
Kaplan-Meier estimates of 3-year all-cause mortality across different flow groups in patients with likely AS-CA (double positivity). Significant differences in survival were observed in patients with likely AS-CA across different flow groups (high-gradient [HG] (black bars), classical low-flow low-gradient [cLFLG] (red bars), paradoxical low-flow low gradient [pLFLG] (green bars) and normal flow low-gradient [NFLG] (blue bars)).AS-CA: aortic stenosis and cardiac amyloidosis.

Follow-up rates at 1, 2, and 3 years were 99%, 98% and 96%, respectively. Within 3 years after TAVI, 817 patients (30%) had died. All-cause mortality of likely AS-CA was 44% (95% confidence interval, CI, 29–45%) compared to 29% (95% CI, 27–31%) among lone AS patients (*p* < 0.001) (Fig. 3). After adjustment for the STS score, hazard ratio (HR) no longer indicated a significant mortality difference between the two groups (HR 1.2 (95% CI, 0.99–1.53), *p* = 0.065). Stratification by AS flow groups revealed no significant difference in mortality between AS lone and likely AS-CA across the HG, cLFLG and NFLG groups, whereas in the pLFLG group, likely AS-CA was associated with a significant increase in mortality risk (Supplementary Fig. 2). In a separate analysis restricted to patients with likely AS-CA, 3-year mortality differed significantly across flow groups, with the highest mortality observed in the low flow groups (*p* = 0.009) (Fig. 4).

Symptomatic benefit from TAVI according to New York Heart Associated class (NYHA) was comparable for all patients (improvement by ≥1 class in 76% (AS lone) and 73% (AS-CA), *p* = 0.588).

## 4 Discussion

To the best of our knowledge, this study represents the largest single-center cohort of patients with severe aortic stenosis (AS) evaluated for the likelihood of concomitant cardiac amyloidosis. [7] Application of two CA likelihood scores, combined with a detailed analysis of their individual components, revealed substantial variability (31%) in the estimated likelihood of AS-CA dual pathology within our cohort. Differences in score composition may partly explain this variability, including the incorporation of ECG parameters and biomarkers in the RAISE score as well as differing thresholds for age (≥85 years vs. ≥80 years) and septal wall thickness (≥18 mm vs. ≥16 mm). Importantly, the T-AMYLO score assigns 3 points to all men and classifies them as “inconclusive” or, if one red-flag criterion is positive, as “likely”, which probably led to the different positivity rates of the two scores.

The RAISE Score, developed by Nitsche et al. [1], was especially designed to identify AS patients with increased likelihood of ATTR-CM and integrates clinical and diagnostic parameters derived from 407 consecutive AS patients across three centers. Proposed cut-off values of ≥2 and ≥ 3 points indicate ATTR-CM with sensitivities of 72–94% and specificities of 52% and 85%, respectively. In the present analysis, we applied a threshold of ≥3 points, as a cut-off of ≥2 points would have resulted in an estimated likelihood of 43% in our cohort, likely overestimating the likelihood of CA. Recent validation studies applying a RAISE score threshold of ≥3 reported estimated AS-CA likelihoods of 12–28%, while confirmatory scintigraphy identified dual pathology in only 6–8% of cases. [8], [9]

In contrast, the T-AMYLO Score was originally developed as a predictive model for ATTR-CM in a non-prespecified cohort. [2] It stratifies patients into low, intermediate (≥3 points), and high probability (≥7 points) categories. Because the simplified score has also been validated in patients with severe AS (AUC 0.87; sensitivity 37%, specificity of 97%), we applied it as a complementary tool alongside the RAISE score that was more sensitive in its design. Within our cohort, only 2% of patients reached the high-probability threshold (≥7 points). Therefore, we used a cut-off of ≥3 points to identify patients requiring further evaluation. However, because male sex contributes substantially to the score, all male patients automatically reach at least 3 points, limiting the discriminatory capacity of this threshold. Consequently, a more comprehensive assessment incorporating extracardiac manifestations, imaging findings, and laboratory parameters was required to refine diagnostic suspicion. Using this approach, 1202 patients (44%) in our cohort would have met criteria warranting further evaluation for possible dual pathology according to the T-AMYLO score.

Given the conceptual overlap between the RAISE and T-AMYLO scores, we subsequently analyzed the subgroup of patients meeting the defined cut-off criteria in both scoring systems. This approach yielded an estimated likelihood of AS-CA dual pathology of approximately 9% within the overall cohort. The requirement for concordant positivity of both scores was intended to increase the robustness of the analysis and to reduce dependence on a single screening tool, rather than to establish a novel validated diagnostic algorithm. The estimated likelihood of 9% was somewhat lower than reported in previous studies and may, at least in part, reflect reporting bias or incomplete documentation of individual scoring variables. [1], [3], [7] In particular, systematic assessment of extracardiac manifestations remains challenging in studies evaluating the coexistence of AS and CA. [7] Basically, likelihood scores should be regarded as individual components of a broader diagnostic framework and interpreted alongside clinical expertise and the overall clinical presentation, particularly in centers experienced in the management of amyloidosis. While they provide a structured approach to estimate the plausibility of a given condition, their reliability depends on the underlying data and is influenced by bias, data quality and pretest probability. [10]

Patients with low-flow, low-gradient AS, including both cLFLG and pLFLG phenotypes, generally have a poorer prognosis than patients with high-gradient AS owing to a higher burden of concomitant cardiac disease, including coronary artery disease and heart failure. This broader disease burden should be considered when interpreting the higher mortality observed in the low-flow, low-gradient subgroups with high estimated AS-CA likelihood. Importantly, many of these comorbidities are reflected in the STS score. Accordingly, the initially apparent increase in mortality was attenuated after STS adjustment, suggesting that the excess risk was largely attributable to the greater burden of underlying comorbidities rather than to the estimated likelihood of AS-CA itself. Despite a significantly higher STS scores at baseline, our data did not indicate an increased periprocedural risk in patients with suspected AS-CA.

Notably, these patients still derived meaningful clinical benefit from TAVI, particularly with respect to symptomatic improvement as reflected by NYHA class.

Our analysis did not show a predominance of any specific AS flow phenotype among patients with a likelihood of AS-CA. While previous reports suggested that dual pathology occurs mainly in patients with paradoxical LFLG AS, this was not observed in our cohort. Our findings support the concept that the coexistence of AS and CA reflects a disease continuum rather than a phenotype restricted to a specific hemodynamic subgroup. Persistent exposure to both untreated conditions may contribute to progressive ventricular dysfunction over time. Mechanistically, chronic pressure overload in AS is associated with extracellular matrix remodeling, low-grade inflammation, and subendocardial ischemia – processes that may promote amyloid fibril deposition and myocardial fibrosis, often progressing along an endocardial-to-epicardial gradient. [11] Moreover, increased mechanical shear stress in AS may facilitate transthyretin cleavage and subsequent amyloid deposition through mechanoenzymatic mechanisms. [12]

In the light of emerging disease-modifying therapies for CA, a pragmatic stepwise diagnostic approach may be warranted. Such an approach could start with an initial clinical risk stratification using likelihood scores such as RAISE and T-AMYLO, including a more structured assessment of extracardiac manifestations and be followed by targeted imaging and confirmatory testing according with current ESC recommendations. [13] The importance of timely identification of concomitant AS-CA is further supported by recent data suggesting that the combination of aortic valve replacement and ATTR-specific therapy is associated with particularly favorable outcomes. [14]

The proposed approach may facilitate earlier recognition of AS-CA among patients referred for TAVI while maintaining a focused and efficient diagnostic workflow in routine AS care.

### 4.1 Limitations

Several limitations of this study should be acknowledged. First, due to the retrospective study design, no systematic diagnostic work-up specifically aimed at confirming CA was performed. Furthermore, red-flag criteria for both scores were applied retrospectively using routinely collected clinical data. Incomplete documentation of individual score components may have affected score calculation and, subsequently, the estimated likelihood of AS-CA. Consequently, the present analysis should be interpreted as an exploratory assessment of disease likelihood rather than a definitive diagnostic evaluation. This also limits generalizability to an unselected TAVI cohort.

Second, the data analyzed for the present study are derived from patients that were referred for TAVI in a period when CA only played a minimal role in clinical routine. The non-invasive diagnostic algorithm for CA introduced in 2021 ESC guidelines was just gradually implemented during the study period. As a result, confirmatory imaging or histological data were not available in a sufficient proportion of patients to allow meaningful subgroup analysis. Additionally, identification of dual pathology had limited therapeutic implications, as disease-modifying stabilizing therapies received regulatory approval in 2020. Accordingly, the present analysis reflects a pre-disease-modifying therapy era and should be interpreted primarily as descriptive and hypothesis-generating. In addition, both likelihood scores were published after patient referral, meaning their application represents a retrospective exploratory analysis rather than a diagnostic strategy implemented in real time.

Third, the scores applied in this study were not originally developed to assess differences across flow groups in AS populations. Additionally, subgroup sizes were limited, particularly for AS-CA cases within individual flow groups. Hence, the absence of statistically significant differences should be interpreted with caution, and a type II error cannot be excluded.

## 5 Conclusion

In conclusion, rates of likely AS-CA dual pathology assessed using two established likelihood scores did not differ across AS flow groups. Although AS-CA likelihood was associated with increased long-term mortality, this difference was attenuated after adjustment for STS score. There were no significant differences in VARC-3 outcomes in AS-CA patients. Importantly, likely AS-CA patients derived a significant symptomatic benefit comparable to those with lone AS. In light of emerging disease-modifying therapies for CA, further studies are warranted to refine diagnostic strategies and improve identification of dual pathology in this particular setting.

## Clinical perspectives

Concomitant transthyretin amyloid cardiomyopathy (CA) is a relevant but heterogeneous comorbidity in patients with severe aortic stenosis (AS). In a large TAVI cohort, the pooled use of two scores identified a lower-than-expected estimated likelihood of AS–CA without enrichment in specific AS flow groups, especially not among paradoxical low-flow, low-gradient AS. Procedural success and symptomatic improvement after TAVI were comparable between patients with isolated AS and those with likely AS–CA.

## CRediT authorship contribution statement

**Stéphanie Kristina Schwarting:** Writing – review & editing, Writing – original draft, Software, Methodology, Formal analysis, Conceptualization. **Selen Alieva:** Resources, Data curation. **Mohammad Usama Chaudhry:** Resources, Data curation. **Kornelia Löw:** Writing – review & editing, Validation, Resources, Formal analysis. **Sergio Grosu:** Writing – review & editing. **Steffen Massberg:** Writing – review & editing, Supervision, Resources, Project administration, Investigation. **Simon Deseive:** Writing – review & editing, Supervision, Resources, Project administration, Investigation. **Julius Steffen:** Writing – review & editing, Visualization, Validation, Supervision, Software, Resources, Project administration, Methodology, Investigation, Formal analysis, Conceptualization.

## Funding

None.

## Declaration of competing interest

The authors declare that they have no known competing financial interests or personal relationships that could have appeared to influence the work reported in this paper.

## Data Availability

The datasets generated and analyzed for the current study are available from the corresponding author upon specific and reasonable request.
