# Supplementary material for: Dual pathology in aortic stenosis: Identifying transthyretin amyloidosis likelihood in patients referred for transcatheter aortic valve implantation
Source: Int J Cardiol Heart Vasc. 2026 Aug 12;66:101991. doi: 10.1016/j.ijcha.2026.101991 (PMC13486978; doi:10.1016/j.ijcha.2026.101991)
Supplement: Supplementary file 1 [file mmc1.docx]

**Supplemental Material**

**Supplemental Table 1 -** Baseline characteristics across aortic stenosis flow groups

|  | Total (N=2764) | HG (N=1309) | cLFLG (N=498) | | pLFLG  (N=517) | NFLG (N=440) | | p value |
| --- | --- | --- | --- | --- | --- | --- | --- | --- |
| Male sex, n | 1531 (55) | 671 (51) | 370 (74) | | 281 (54) | 209 (48) | | < 0.01 |
| Age, years | 81.8 [77.6, 85.4] | 81.2 [76.8, 85.1] | 82.3 [78.2, 86.0] | | 82.5 [78.5, 85.7] | 81.8 [78.4, 85.2] | | < 0.01 |
| Body mass index, kg/m^2^ | 25.8 [23.4, 29.0] | 25.9 [23.5, 29.4] | 25.5 [23.1, 28.4] | | 26.0 [23.8, 29.3] | 25.4 [23.0, 28.3] | | < 0.01 |
| Body surface area, m^2^ | 1.9 [1.7, 2.0] | 1.9 [1.7, 2.0] | 1.9 [1.8, 2.0] | | 1.9 [1.7, 2.0] | 1.8 [1.7, 1.9] | | < 0.01 |
| STS score, points | 3.0 [2.0, 5.0] | 3.0 [2.0, 4.2] | 4.7 [2.8, 7.5] | | 3.4 [2.0, 5.8] | 3.0 [2.0, 5.0] | | < 0.01 |
| Hypertension | 2481 (90) | 1154 (89) | 451 (91) | | 476 (93) | 400 (91) | | 0.07 |
| Type 2 DM, n | 756 (28) | 338 (26) | 161 (33) | | 155 (30) | 102 (23) | | < 0.01 |
| Smoker (active or past), n | 659 (24) | 299 (23) | 147 (30) | | 117 (23) | 96 (22) | | 0.01 |
| COPD, n | 208 (12) | 71 (9) | 48 (15) | | 42 (14) | 47 (17) | | < 0.01 |
| Hypercholesterolaemia, n | 1414 (52) | 649 (50) | 248 (51) | | 274 (54) | 243 (56) | | 0.21 |
| CKD, n | 1062 (38) | 402 (31) | 297 (60) | | 219 (42) | 144 (33) | | < 0.01 |
| Creatinine, mg/dl | 1.1 [0.9, 1.4] | 1.0 [0.9, 1.3] | 1.3 [1.0, 1.6] | | 1.1 [0.9, 1.4] | 1.0 [0.9, 1.3] | | < 0.01 |
| eGFR, ml/min/1.73m^2^ | 62 [44, 79] | 66 [47, 81] | 51 [33, 70] | | 59 [43, 76] | 68 [51, 83] | | < 0.01 |
| CAD, n | 1586 (57) | 674 (52) | 338 (70) | | 313 (61) | 261 (60) | | < 0.01 |
| Prior MI, n | 290 (11) | 90 (7) | 105 (21) | | 52 (10) | 43 (10) | | < 0.01 |
| hsTroponin T, ng/l | 27 [16, 48] | 25 [15, 41] | 48 [28, 83) | | 24 (16, 41) | 22 (14, 35) | | < 0.01 |
| NTproBNP, pg/ml | 1697 [627, 4344] | 1451 [568, 3671] | 5369 [2442, 11786] | | 1798 [721, 3581] | 731 [358, 1722] | | < 0.01 |
| Echocardiographic parameters | | | |  | | |  | |
| LVEF, % | 55 [49, 55] | 55 [52, 55] | 40 [32, 45] | | 55 [55, 55] | 55 [55, 55] | | < 0.01 |
| IVSd, mm | 13 [11, 15] | 13 [12, 15] | 12 [11, 14] | | 13 [11, 14] | 13 [11, 14] | | < 0.01 |
| LVPWd, mm | 11 [10, 13] | 12 [10, 14] | 11 [10, 13] | | 11 [10, 13] | 11 [10, 13] | | < 0.01 |
| RWT, % | 0.5 [0.4, 0.6] | 0.5 [0.4, 0.6] | 0.4 [0.4, 0.5] | | 0.5 [0.4, 0.6] | 0.5 [0.4, 0.6] | | < 0.01 |
| LVEDd, mm | 47 [42, 52] | 46 [41, 51] | 52 [46, 57] | | 45 [40, 50] | 45 [41, 50] | | < 0.01 |
| E/A | 0.8 [0.6, 1.3] | 0.8 [0.6, 1.1] | 1.2 [0.8, 1.8] | | 0.8 [0.8, 1.5] | 0.8 [0.6, 1.1] | | <0.01 |
| E’ | 6.2 [5.0, 7.7] | 6.0 [4.9, 7.5] | 5.9 [4.8, 7.0] | | 7.3 [5.9, 8.6] | 6.1 [5.1, 7.4] | | <0.01 |
| E/e’ | 14.1 [10.6, 18.5] | 14.2 [10.8, 18.4] | 15.6 [11.7, 21.2] | | 13.2 [10.0, 16.9] | 13.9 [10.4, 17.9] | | <0.01 |
| AVA, cm^2^ | 0.7 [0.6, 0.8] | 0.7 [0.5, 0.8] | 0.7 [0.6, 0.8] | | 0.7 [0.6, 0.8] | 0.9 [0.8, 0.9] | | < 0.01 |
| AVAi, cm^2^/m^2^ | 0.4 [0.3, 0.5] | 0.4 [0.3, 0.4] | 0.4 [0.3, 0.4] | | 0.4 [0.3, 0.4] | 0.5 [0.4, 0.5] | | < 0.01 |
| Dpmax, mmHg | 61 [47, 74] | 75 [68, 86] | 42 [32, 52] | | 45 [38, 54] | 54 [46, 60] | | < 0.01 |
| Dpmean, mmHg | 39 [28, 47] | 47 [43, 54] | 26 [20, 31] | | 28 [22, 34] | 33 [28, 36] | | < 0.01 |
| SVi | 34 [28, 42] | 38 [31, 45] | 27 [22, 32] | | 29 [25, 32] | 41 [37, 45] | | < 0.01 |
| AI >1 | 313 (11) | 166 (13) | 64 (13) | | 44 (9) | 39 (9) | | 0.02 |
| TI >1 | 395 (15) | 135 (10) | 111 (23) | | 103 (20) | 46 (11) | | < 0.01 |
| TAPSE, mm | 21 [18, 24] | 22 [19, 25] | 18 [14, 21] | | 20 [17, 23] | 23 [20, 26] | | < 0.01 |
| TR gradient, mmHg | 33 [26, 43] | 33 [25, 44] | 37 [29, 45] | | 34 [27, 43] | 31 [25, 40] | | 0.01 |
| MR grade ≥ 3, n | 108 (4) | 40 (3) | 42 (9) | | 19 (4) | 7 (2) | | < 0.01 |
| RA area, mm^2^ | 20.1 [16.5, 25.1] | 19.3 [15.7, 23.3] | 23.1 [18.9, 28.4] | | 21.3 [17.6, 27.1] | 19.1 [15.8, 22.9] | | < 0.01 |
| LA area, mm^2^ | 23.8 [19.8, 28.4] | 23.3 [19.4, 27.6] | 25.6 [21.6, 30.6] | | 24.5 [20.2, 30.1] | 22.5 [18.9, 26.4] | | < 0.01 |

Data are presented as *n* (%) or median (IQR). A *p* value of < 0.05 was considered significant.

Afib/flutter: atrial fibrillation/atrial flutter; AR: aortic regurgitation; AVA: aortic valve area; AVAi: aortic valve area index; dpmax: maximum pressure gradient; dpmean: mean pressure gradient; CAD: coronary artery disease; CKD: chronic kidney disease; cLFLG: classical low-flow, low-gradient flow group; COPD: chronic obstructive pulmonary disease; DM: diabetes mellitus; eGFR: estimated glomerular filtration rate; HG: high-gradient flow group; hs Troponin T: high-sensitive troponin T; IVSd: interventricular septum diastolic diameter; LA: left atrium; LFLG: low-flow, low-gradient; LVEF: left-ventricular ejection fraction; LVIDd left ventricular internal diastolic diameter; LVPWd left ventricular posterior wall diastolic diameter; MI: myocardial infarction; MR: mitral regurgitation; NFLG: normal-flow, low-gradient flow group; NT-proBNP: N-terminal pro brain-natriuretic peptide; pLGLF: paradoxical low-flow, low-gradient flow group; RA: right atrium; RV-PA: right-ventricular to pulmonary artery coupling; RWT: relative wall thickness; STS-Score: Society of Thoracic Surgeon (STS) score; SV: stroke volume; SVi: stroke volume index; TAPSE; tricuspid annular plane systolic excursion; TR: tricuspid regurgitation.

**Supplementary Figure 1 - Distribution of individual score components in patients with severe aortic stenosis.**

Relative frequency of individual variables included in the RAISE score (A) and in the T-AMYLO score (B). Bars represent the proportion of patients fulfilling each criterion among all patients (green), patients meeting the respective score threshold (RAISE ≥3 or T-AMYLO ≥ 3; purple), and patients classified as likely AS-CA based on double positivity in both scores (blue). Variables included voltage criteria, E/A ratio, interventricular septal thickness (IVSd), high-sensitivity troponin T (hsTropT), age thresholds, right bundle branch block (RBBB), carpal tunnel syndrome (CTS), and sex, as illustrated in Figure 1. Percentages are displayed relative to the respective subgroup sizes.

**Supplemental Figure 2 – 3-year all-cause mortality across aortic stenosis flow groups in patients with AS lone and likely AS-CA.**

Stratification by AS flow groups revealed no significant differences in mortality between AS lone and likely AS-CA across high-gradient (HG, panel A), classical low-flow, low-gradient AS (cLFLG, panel B) and normal-flow, low-gradient AS (NFLG, panel D), whereas in paradoxical low-flow, low-gradient (pLFLC, panel C) group, likely AS-CA was associated with significant increase in mortality risk.
